# Shock index and heart rate standard reference values in the immediate postpartum period: A cohort study

**DOI:** 10.1371/journal.pone.0217907

**Published:** 2019-06-11

**Authors:** Anderson Borovac-Pinheiro, Filipe Moraes Ribeiro, Sirlei Siani Morais, Rodolfo Carvalho Pacagnella

**Affiliations:** Department of Obstetrics and Gynaecology, School of Medical Sciences, University of Campinas, Campinas (SP), Brazil; Monash University, AUSTRALIA

## Abstract

**Objective:**

To determine Shock Index (SI) reference values in the first two hours of the postpartum period after objectively measuring postpartum bleeding.

**Materials and methods:**

A complementary analysis using data from a prospective cohort study at Women’s Hospital of the University of Campinas, Brazil, between 1 February 2015 and 31 March 2016. It included women giving birth vaginally unless they had one of these conditions: gestational age below 34 weeks, hypertension, hypo- or hyperthyroidism without treatment, any cardiac disease, infections with fever or sepsis, history of coagulopathy or delivery by C-section. Blood loss was measured by adding the blood volume collected in the drape placed under the women’s buttocks and the weight of gauzes and compresses used (excluding the dry weight). Vital signs were measured every 5–15 min after delivery. Exploratory data analysis was performed to assess the mean, standard deviation, median, and percentiles (5^th^, 10^th^, 25^th^, 50^th^, 75^th^, 90^th^, 95^th^). To identify variation among the periods after delivery, the mean SI and heart rate (HR) values observed for the following intervals were used in the analysis: 0–20 min, 21–40 min, 41–60 min, 61–90 min and 91–120 min.

**Results:**

One hundred eighty-six women were included. The mean age ± SD was 24.9 ± 6.1 years and the mean gestational age at birth was 39.2 ± 1.8 weeks. At the puerperal period, the mean SI values ranged from 0.68 ± 0.14 to 0.74 ± 0.15. The percentile distribution ranged from 0.46 (5^th^ percentile) to 1.05 (95^th^ percentile). The mean HR values ranged from 80.8 ± 12.7 bpm to 92.3 ± 14.4 bpm. The percentile distribution ranged from 62.0 bpm (5^th^ percentile) to 117 bpm (95^th^ percentile).

**Conclusion:**

Reference ranges were established for SI and HR values which showed small variations throughout the postpartum period.

## Introduction

Bleeding associated with childbirth and the postpartum period is the leading cause of postpartum maternal death and morbidity worldwide [1]. In 2015, the worldwide postpartum maternal mortality rate was 195 deaths per 100,000 live births, while in Brazil the estimated rate was 65 deaths per 100,000 live births [1]. About 80% of postpartum maternal deaths in low-income countries are related to postpartum haemorrhage (PPH) [1]. PPH also plays a significant role in maternal morbidity related to ICU patient admission, massive transfusion of blood products, and renal and respiratory failure [2,3].

Obstacles to decreasing these numbers are mainly related to difficulties in the prevention and diagnosis of PPH. Prediction models that consider several risk factors for PPH can only predict excessive bleeding in 10% of cases [4]. There is currently no accurate method for the diagnosis of PPH, and the methods used do not correlate with the severity of blood loss and maternal prognosis. The World Health Organisation recommends a visual estimation of blood loss (VEBL) as a way to quantify the volume lost during childbirth and the postpartum period since it is easier and more convenient in the different contexts of clinical practice [4,5]. However, VEBL is imprecise and tends to overestimate small losses and to underestimate large losses; therefore, the use of blood loss criteria to identify PPH may delay appropriate interventions [6,7].

There is a trend to study cardiovascular physiology to identify strategies for early recognition of PPH [8]. Considering the cardiovascular changes, the Shock Index (SI) is being suggested as a possible indicator of PPH. It is calculated by dividing heart rate by systolic blood pressure (HR/SBP). Previous studies have shown that SI correlated well with gestational blood loss and is apparently a valuable and sensitive tool for haemodynamic changes, rather than classic vital signs such as heart rate and systolic or diastolic blood pressure [9].

Reference values for postpartum SI are not well-established [10,11]. The few studies that have considered SI relied on visual estimation of blood loss, which may not be reliable [10,11] and focused on vital signs recorded immediately after delivery [11] or within the first hour [10]. There remains a lack of well-conducted studies comparing variations in SI in the first two hours following delivery and when blood loss is measured objectively. Therefore, we aimed to determine Shock Index reference values in the first two hours of the postpartum period through a complementary analysis of a prospective cohort study that objectively measured postpartum bleeding.

## Materials and methods

We performed a complementary analysis using data from a prospective cohort study that we conducted at Women’s Hospital of the University of Campinas, Brazil, between 1 February 2015 and 31 March 2016. In the cohort study, we included all women giving birth vaginally unless they had one of these conditions: gestational age below 34 weeks, hypertension, hypo- or hyperthyroidism without treatment, any cardiac disease, infections with fever or sepsis, history of coagulopathy, or postpartum bleeding greater than 500 mL.

We invited women to participate after providing information about the study; if they accepted, they signed an informed consent form. The Institutional Review Board approved the study (CAEE: 26787114.3.0000.5404).

Immediately after delivery, we placed a calibrated drape under the woman’s buttocks to measure blood loss (BRASSS_V drape). We calculated blood loss by adding the blood volume collected in the drape and the weight of gauzes and compresses used (excluding the dry weight). Vital signs were measured every 5 min during the period the woman was in birth position (including, for instance, the period when lacerations were sutured); after this, they were measured every 15 min for 2 hours after delivery. We assumed blood density as 1g/mL [12]. All women prophylactically received 10 IU of oxytocin IV after birth as institutional protocol.

We selected all women who bled less than 500 mL within 2 hours after delivery and performed statistical analysis with the vital signs collected during the period.

Shock Index (SI) was calculated by dividing heart rate (HR) by systolic blood pressure (SBP). Exploratory data analysis was performed to assess the mean, standard deviation, median, and percentiles (5^th^, 10^th^, 25^th^, 50^th^, 75^th^, 90^th^, 95^th^). To identify variation among the periods after delivery, the mean of SI and HR values observed for the following intervals were used in the analysis: 0–20 min, 21–40 min, 41–60 min, 61–90 min and 91–120 min. The differences in SI and HR among the postpartum periods were evaluated by analysis of variance (ANOVA) with the post-hoc Tukey test for independent measurements. ANOVA and Tukey tests were used to assess changes in SI values according to Body Mass Index (BMI) and maternal age.

We defined a significance level of 5% for all analyses and used SAS 9.4.

FAPESP and Faepex–Unicamp funded this research although playing no role in the design and interpretation of the results.

## Results

Among the 270 women in the cohort study, 186 had blood loss less than 500 mL within 2 hours after birth and were included in the present study. Sociodemographic and obstetric characteristics are described in Table 1. The mean age ± SD was 24.9 ± 6.1 years and the mean gestational age at birth was 39.2 ± 1.8 weeks.

**Table 1:** Sociodemographic and obstetric characteristics of women.

Characteristics	n	Mean ± SD
**Age**	**186**	**24.9 ± 6.1**
**BMI**	**171**	**29.1 ± 6.1**
**Parity**	**186**	**0.90 ± 1.17**
**Gestational age**	**186**	**39.2 ± 1.8**
**Education (years)**	**156**	**9.8 ± 2.7**
**Characteristics**	**n (%)**	** **
Ethnicity*		
**White**	**121 (66.1%)**	
**Non-white**	**65 (33.9%)**	
**Onset of labor**		
**Spontaneous**	**132 (71%)**	
**Induced**	**54 (29%)**	
**Anaesthesia**		
**None**	**38 (20.5%)**	
**Spinal anaesthesia**	**148 (79.5%)**	
**Mode of delivery**		
**Vaginal**	**181 (97.3%)**	
**Forceps**	**7 (3.7%)**	
**Episiotomy**		
**No**	**131 (69.4%)**	
**Yes**	**55 (30.6%)**	** **

*missing 7

In the puerperal period, the mean SI values ranged from 0.68 ± 0.14 to 0.74 ± 0.15. The percentile distribution ranged from 0.46 (5^th^ percentile) to 1.05 (95^th^ percentile). There is a trend of decreasing SI values from the “0–20 min” period to “91–120 min” period; nevertheless, there are no statistical differences in SI values among the periods of 21–40 min, 41–60 min, 61–90 min and 91–120 min (Tukey test).

**Fig 1:**
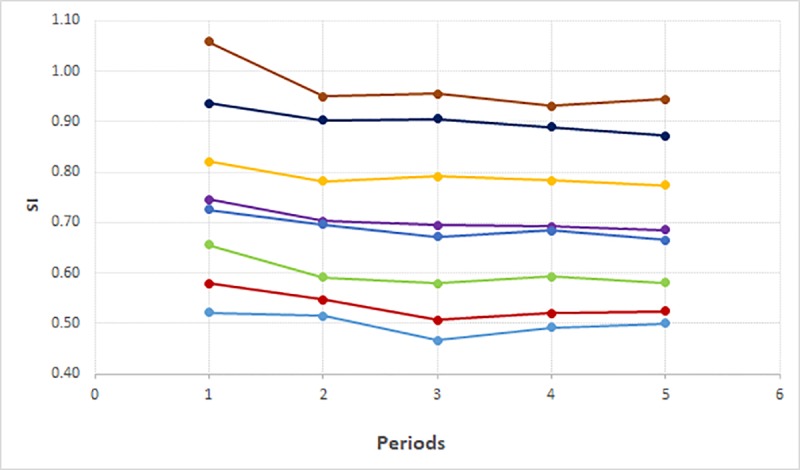
Graph showing percentile Shock Index values according to the period after delivery (SI = Shock Index in bmp/mmHg. Periods: 1 = 0–20 min, 2 = 21–40 min, 3 = 41–60 min, 4 = 61–90 min, 5 = 91–120 min. Percentiles: p5;p10;p25;p50;mean;p75;p90;p95.

**Fig 2:**
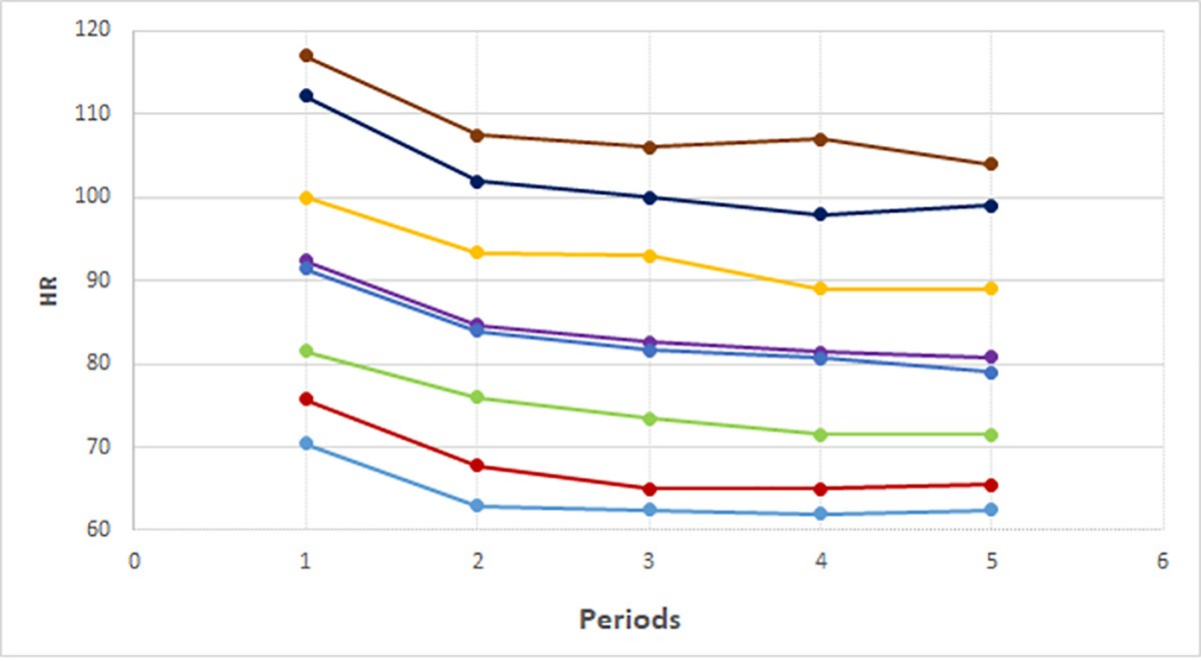
Graph showing percentile heart rate values according to the period after delivery (HR = heart rate in bmp). Periods: 1 = 0–20 min, 2 = 21–40 min, 3 = 41–60 min, 4 = 61–90 min, 5 = 91–120 min. Percentiles: p5;p10;p25;p50;mean;p75;p90;p95.

The mean HR values ranged from 80.8 ± 12.7 bpm to 92.3 ± 14.4 bpm. The percentile distribution ranged from 62.0 (5^th^ percentile) to 117 bpm (95^th^ percentile). The same trending for SI was observed with higher values within 20 min after birth and lower values between 91 and 120 min after delivery. The mean, SD and percentiles of SI and HR are shown in Table 2. Fig 1 shows the distribution of values by percentiles of SI according to the period after delivery and Fig 2 shows the same distribution for HR.

**Table 2:** Mean, standard deviation and percentiles of SI and HR within 2 hours after birth.

Shock Index	**n = 186**	**Mean ± SD**^a^	**Shock Index by Percentile**						
	** **	** **	**5**^**th**^	**10**^**th**^	**25**^**th**^	**50**^**th**^	**75**^**th**^	**90**^**th**^	**95**^**th**^
	**0–20 min**	0.74 ± 0.15	0.52	0.58	0.65	0.72	0.82	0.93	1.05
	**21–40 min**	0.70 ± 0.14 ^b^	0.51	0.54	0.59	0.69	0.78	0.90	0.95
	**41–60 min**	0.69 ± 0.15 ^b^^,^^c^	0.46	0.50	0.58	0.67	0.79	0.90	0.95
	**61–90 min**	0.69 ± 0.14 ^b^^,^^c^	0.49	0.52	0.59	0.68	0.78	0.88	0.93
	**91–120 min**	0.68 ± 0.14 ^b^^,^^c^	0.50	0.52	0.58	0.66	0.77	0.87	0.94
Heart Rate	**n = 186**	**Mean ± SD**	**Heart Rate by Percentile**						
	** **	** **	**5**^**th**^	**10**^**th**^	**25**^**th**^	**50**^**th**^	**75**^**th**^	**90**^**th**^	**95**^**th**^
	**0–20 min**	92.3 ± 14.4	70.4	75.8	81.5	91.4	100.0	112.2	117.0
	**21–40 min**	84.8 ± 13.5 ^b^	63.0	67.7	76.0	84.0	93.3	102.0	107.5
	**41–60 min**	82.6 ± 13.4 ^b^^,^^c^	62.5	65.0	73.5	81.7	93.0	100.0	106.0
	**61–90 min**	81.4 ± 13.3 ^b^^,^^d^	62.0	65.0	71.5	80.8	89.0	98.0	107.0
	**91–120 min**	80.8 ± 12.7 ^b^^,^^c^	62.5	65.5	71.5	79.0	89.0	99.0	104.0

a The variation in SI values was significant by ANOVA (P < 0.001).

b Significant difference from 0–20 min by Tukey test (P < 0.05)

c No significant difference among the other moments (P > 0.05)

d Significant difference from 21–40 min by Tukey test (P < 0.05)

Table 3 illustrates the mean and standard deviation of SI stratified by BMI group and maternal age. The group with a BMI of 30 or more had the lowest mean SI values compared with the 19–24 and 25–29 groups. Nevertheless, statistical differences were found between the groups with BMI 19–24 vs ≥ 30 at 0–20 min and the groups BMI 25–29 vs ≥ 30 at the periods: 21–40 min, 41–60 min and 61–90 min. No differences were found among maternal age groups.

**Table 3:** Mean and standard deviation of SI by BMI group and maternal age.

	**BMI Group**			
	**19–24 kg/m**^**2**^	**25–29 kg/m**^**2**^	**≥ 30 kg/m**^**2**^	
	**Mean ± SD**	**Mean ± SD**	**Mean ± SD**	**p-value**
**0–20 min**^a^	0.78 ± 0.16	0.76 ± 0.14	0.70 ± 0.14^b^	0.02
**21–40 min**^a^	0.72 ± 0.15	0.73 ± 0.14	0.66 ± 0.12^c^	0.03
**41–60 min**^a^	0.71 ± 0.17	0.72 ± 0.15	0.66 ± 0.14^c^	0.04
**61–90 min**^a^	0.69 ± 0.16	0.73 ± 0.14	0.66 ± 0.11^c^	0.02
**91–120 min**	0.69 ± 0.14	0.71 ± 0.15	0.65 ± 0.12	0.06
	**Maternal Age**			
	**≤ 19y**	**20–34y**	**≥ 35y**	
	**Mean ± SD**	**Mean ± SD**	**Mean ± SD**	**p-value**
**0–20 min**	0.77 ± 0.16	0.74 ± 0.15	0.70 ± 0.12	0.24
**21–40 min**	0.72 ± 0.16	0.70 ± 0.14	0.65 ± 0.01	0.41
**41–60 min**	0.70 ± 0.18	0.70 ± 0.14	0.62 ± 0.17	0.23
**61–90 min**	0.70 ± 0.16	0.70 ± 0.13	0.62 ± 0.12	0.26
**91–120 min**	0.67 ± 0.15	0.69 ± 0.13	0.61 ± 0.13	0.15

a The variation in SI values was significant by ANOVA (P < 0.05).

b Significant difference from 19–24 by Tukey test (P<0.05)

c Significant difference from 25–29 by Tukey test (P<0.05)

## Discussion

Our study aimed to evaluate the range of SI and HR values within 2 hours after delivery. The data showed that SI and HR values were higher within 20 min after delivery and tended to decrease over time. The values were influenced by BMI, yet maternal age does not show influence among the values founded.

Within 20 min after birth, HR and therefore SI may be higher due to the influence of confounding factors such as pain, pushing efforts, anxiety and the emotion of seeing the baby for the first time. It is influenced by the rapid postpartum elevation of cardiac output (up to 60–80% increase) [13]. Ten minutes after delivery cardiac output decreases rapidly, to values approaching normal, one hour after birth [13]. After the first 20 min, the HR and SI are more stable, with no statistical differences among the studied periods.

A previous study by our group demonstrated reference standard values for SI during pregnancy [14]. For gestational ages between 33–36 weeks and above 37 weeks, the mean ± SD SI values found were 0.82 ± 0.14 and 0.79 ± 0.13 respectively. These values are slightly higher than the mean ± SD (0.70 ± 0.14) found in the present study after 20 min of birth.

The actual diagnostic method to identify women with postpartum haemorrhage (PPH) is based on visual estimation of blood loss [7]. Nevertheless, the accuracy of the visual estimation and the actual definition of PPH have been questioned [8]. It has been suggested that the inclusion of changes in vital signs, particularly the SI, could be used as an adjuvant tool for the early identification of women with PPH [8].

Some studies have shown the relationship between increased SI values and severe postpartum outcomes related to PPH [15–20]. In the previous studies, the SI values above 0.9 in the postpartum period were related to blood loss above 1500 mL, UCI admission [18,19], and massive transfusion [17,18]. Statistically significant higher SI values were found in women that received blood transfusion due to PPH after vaginal delivery [15].

Although some studies have shown SI values are related to adverse outcomes, few studies have established the standard reference values of SI during the postpartum period. The idea is to determine the normal SI values to identify changes in SI after birth that can be used as a tool to recognise women at potential risk and to establish prompt treatment. Taylor et al. found values for SI ranging from 0.46 to 1.07 within 24 hours after delivery [10]. They also found that SI values remained stable within 24 hours after vaginal delivery [10]. Nathan et al. have shown a median SI postpartum value of 0.66, while the lower and upper quartile were 0.6 and 0.74, respectively [11]. In our study, the mean SI at the lower quartile ranged from 0.58 to 0.65 while at the upper quartile ranged from 0.77 to 0.82. The differences found among our and the other studies can be explained due to the different methods used. While in our study we objectively measured blood loss, the others used the visual estimation of blood loss. And while in our study we measured vital signs consistently within 2 hours after delivery, Nathan et al. used the first vital sign measured after delivery for the statistical analysis. Taylor et al. measured vital signs within 1 hour postpartum.

Heart rate is also a vital sign that is related to postpartum bleeding, and it is important to show standard values for HR after delivery. In our study, the HR values ranged from 80.8 ± 12.7 bpm to 92.3 ± 14.4 bpm after the delivery. Nevertheless, while the SI has a trend to stabilise after 21 min after delivery, the HR showed some variations that could cause difficulty with application in daily clinical practice. Nathan et al. found similar values for HR in the postpartum period, with the median of 81 bpm and the lower and upper quartiles of 74 bpm and 88 bpm respectively [11].

Our study has some limitations because it is a complementary analysis using data from a prospective cohort study. But our study also has strengths. Considering that Brazilian women are very heterogeneous and multiethnic, our results can be applied to women worldwide. We objectively measured blood loss after delivery and could identify the women who truly bled less than 500 mL after delivery. Besides that, we systematically measured vital signs and divided the results into periods that can be used in clinical practice.

Considering that vital signs (mainly SI and HR) are being suggested to assist in the diagnosis of PPH, more robust data regarding the references of vital signs during the postpartum period need to be defined. Our study brings valuable information about the reference values of SI and HR in the postpartum period. Future prospective studies should evaluate the real role of SI as an adjuvant tool for PPH and include reference standard values for vital signs in women with some medical conditions such as hypertension, cardiomyopathy, anaemia, and hypo- or hyperthyroidism.

## Supporting information

S1 Table Mean, standard deviation and percentiles of SI and HR within 2 hours after birth. (XLSX)
