# Correction: Shock index and heart rate standard reference values in the immediate postpartum period: A cohort study

**DOI:** 10.1371/journal.pone.0230530

**Published:** 2020-03-12

**Authors:** Anderson Borovac-Pinheiro, Filipe Moraes Ribeiro, Sirlei Siani Morais, Rodolfo Carvalho Pacagnella

The following information is missing from the Funding statement: This study was funded by Fundo de Apoio ao Ensino, à Pesquisa e Extensão, Universidade Estadual de Campinas grant PAPDIC/2714 to RCP and Fundação de Amparo à Pesquisa do Estado de São Paulo, grant 2018/06325-2, to ABP.
